# Treatment-free remission after a second TKI discontinuation attempt in patients with Chronic Myeloid Leukemia re-treated with dasatinib – interim results from the DAstop2 trial

**DOI:** 10.1038/s41375-024-02145-6

**Published:** 2024-01-26

**Authors:** Hjalmar Flygt, Stina Söderlund, Johan Richter, Susanne Saussele, Perttu Koskenvesa, Leif Stenke, Satu Mustjoki, Andreja Dimitrijevic, Jesper Stentoft, Waleed Majeed, Lydia Roy, Dominik Wolf, Arta Dreimane, Bjørn Tore Gjertsen, Tobias Gedde-Dahl, Erik Ahlstrand, Berit Markevärn, Henrik Hjorth-Hansen, Jeroen Janssen, Ulla Olsson-Strömberg

**Affiliations:** 1 Department of Medical Science and Division of Hematology, Uppsala University Hospital, Uppsala, Sweden; 2 Department of Hematology, Oncology and Radiation Physics, Skåne University Hospital, Lund, Sweden; 3 Medical Clinic, Medical Faculty Mannheim of the University of Heidelberg, Mannheim, Germany; 4 Department of Hematology, Hematology Research Unit Helsinki and Helsinki University Hospital Comprehensive Cancer Center, Helsinki, Finland; 5 Department of Hematology, Karolinska University Hospital and Department of Medicine, Karolinska Institutet, Stockholm, Sweden; 6 Translational Immunology Research Program and Department of Clinical Chemistry and Hematology, University of Helsinki, Helsinki, Finland; 7 ICAN Digital Precision Cancer Medicine Flagship, Helsinki, Finland; 8 Department of Hematology, Odense University Hospital, Odense, Denmark; 9 Department of Hematology, Aarhus University Hospital, Aarhus, Denmark; 10 Department of Hemato-Oncology, Stavanger University Hospital, Stavanger, Norway; 11 French CML group Fi-LMC, Centre Léon Bérard, Lyon, Hôpital Universitaire Henri Mondor, AP-HP, Service d’hématologie Clinique & Faculté de Santé, Université Paris Est Créteil, Créteil, France; 12 Department of Hematology and Oncology, Comprehensive Cancer Center Innsbruck (CCCI), Tyrolean Cancer Research Institute (TKFI), Medical University Innsbruck, Innsbruck, Austria; 13 Medical Clinic 3, Universitätsklinikum, Bonn, Germany; 14 Department of Hematology, Linköping University Hospital, Linköping, Sweden; 15 Department of Internal Medicine, Hematology Section, Haukeland University Hospital, Bergen, Norway; 16 Department of Hematology, Oslo University Hospital, Rikshospitalet, Oslo, Norway; 17 Department of Medicine, Faculty of Medicine and Health, Örebro University, Örebro, Sweden; 18 Department of Hematology, Umeå University Hospital, Umeå, Sweden; 19 Department of Hematology, St. Olavs Hospital, Trondheim, Norway; 20 Department of Hematology, Radboud University medical center, Nijmegen, The Netherlands

**Keywords:** Myeloproliferative disease, Targeted therapies

## Abstract

Tyrosine kinase inhibitor (TKI) discontinuation in chronic myeloid leukemia (CML) has become part of routine care for patients with a sustained deep molecular response (DMR). Approximately 50% experience a molecular relapse upon TKI cessation. Most of them quickly regain DMR upon TKI resumption. Whether these patients can achieve a second treatment-free remission (TFR) remains unclear. DAstop2 (ClinicalTrials.gov ID: NCT03573596) is a prospective study including patients with a failed first TFR attempt re-treated with any TKI for ≥ one year. Upon entering the study, patients received the TKI dasatinib for additional two years. Patients with sustained DMR for ≥1 year qualified for a second TKI stop. Ninety-four patients were included between Oct 2017-Dec 2021. At the time of data analysis, 62 patients had attempted a 2^nd^ stop. After a median follow-up of 27 months from 2^nd^ stop, TFR rates were 61, 56 and 46% at 6, 12 and 24 months respectively. No progression to advanced stage disease was seen and 87% had re-achieved MR^4^ within a median of 3 months from TKI re-initiation. In summary, we show that a 2^nd^ TFR attempt after dasatinib treatment is safe, feasible and TFR rates seem in the range of those reported in trials of a first TKI stop.

## Introduction

Chronic myeloid leukemia (CML) is characterized by the constitutively active tyrosine kinase BCR::ABL1, and standard therapy is treatment with tyrosine kinase inhibitors (TKI). Not only do patients with an optimal response to TKI treatment today have a life expectancy close to that of an age-matched general population, but a proportion of patients can also successfully discontinue treatment without relapse [1–3]. The feasibility of TKI discontinuation was first demonstrated in a small pilot study followed by the multi-center STIM study where approximately 40% of patients with stable (more than two years) undetectable *BCR::ABL1* transcripts measured by reverse transcriptase quantitative polymerase chain reaction (RT-qPCR) could discontinue imatinib treatment without molecular relapse [4–6]. Later, the study A-STIM established loss of major molecular response (MMR, or MR^3^) as a safe and feasible criterion to re-initiate TKI therapy [7]. Subsequently, many discontinuation trials have demonstrated that discontinuation after first- and second-line 2^nd^ generation TKI is equally safe, and standardized criteria for TKI discontinuation have recently been adopted by guidelines for routine clinical care [7–16]. The highly interdependent variables duration of TKI treatment and duration of deep molecular response (DMR) prior to discontinuation have repeatedly been associated with TFR, including in EURO-SKI, the largest TKI discontinuation study to date [17, 18]. Another consistent finding in all discontinuation studies was that the majority of relapses occurred within the first 6 months upon TKI discontinuation [4, 11–13, 17]. In general, attempting a TFR has been considered safe since large discontinuation trials reported no cases of progression to advanced stage disease. However, very rare cases of progression to blast crisis have been reported [19]. Patients with a molecular relapse after TKI discontinuation generally regain their previously achieved DMR upon TKI re-introduction, presenting a rationale for a second TKI discontinuation attempt. A few retrospective studies such as the French RE-STIM study have already evaluated a second TFR attempt. In this largest study 64% of patients experienced loss of MMR after a median 38 months of follow-up upon the second TKI discontinuation attempt [20, 21].

There is yet no direct comparison between 1^st^ and 2^nd^ generation TKI with regards to the proportion of patients achieving continuous TFR. However, 2^nd^ generation TKIs like dasatinib as up-front treatment of CML have stronger in-vitro kinase inhibitory activity and are efficacious in patients with previous treatment failure on imatinib with a larger proportion of patients achieving DMR [22–25]. We therefore hypothesized that the use of dasatinib could increase the probability of a successful second TKI stop attempt in patients who failed a previous first TFR attempt. A smaller prospective study in 22 CML patients attempting a second TKI discontinuation after re-challenge with 2^nd^ generation TKI nilotinib was recently published, showing that 55% lost MMR after a median 8 months of follow up [26]. The DAstop2 study, an ongoing collaborative study, was designed to evaluate TFR rates after discontinuation of dasatinib in patients with a previous failed TFR attempt. Interim results of this study are presented here.

## Methods

### Study design and study population

**Fig. 1:**
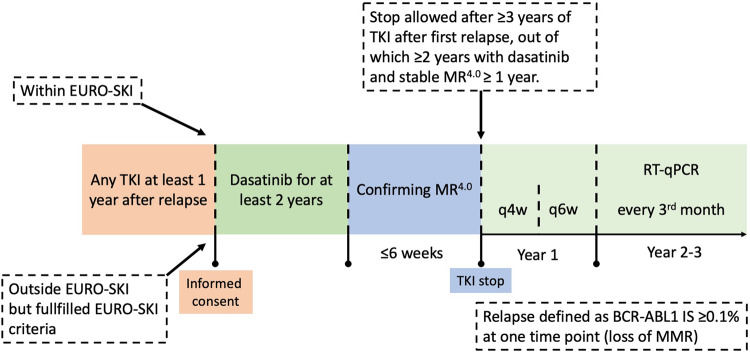
Study outline. Patients with a previous failed discontinuation attempt within EURO-SKI or according to EURO-SKI criteria were eligible for inclusion. After inclusion, patients were switched to dasatinib for a minimum of two years. Patients with ≥3 years of TKI treatment and stable MR^4^ or better for ≥1 year were eligible for a second discontinuation attempt.

Patients were included at 17 university or regional hospitals in Sweden, Denmark, Norway, Finland, the Netherlands, Germany, and France. Patients were eligible if they 1) relapsed in the EURO-SKI trial, or 2) discontinued TKI treatment outside of EURO-SKI and discontinued according to EURO-SKI criteria. Post-relapse, all patients were retreated with any TKI for minimally one year before study inclusion. All patients gave written informed consent. Patients with a hematological relapse after the first TKI stop or who had restarted TKI therapy prematurely without loss of MMR where not eligible for inclusion. Full inclusion and exclusion criteria are presented in a supplementary table (Supplementary Table S3). Upon inclusion, patients were switched to dasatinib 100 mg daily, or 70 mg at the discretion of the investigator, and subsequently treated for two years. For patients already on dasatinib at inclusion, but with a lower dose adjusted due to intolerance (i.e. 20–50 mg), this lower dose was continued. If MR^4^ was re-achieved and maintained for at least one year, at month 24 patients were eligible for a second discontinuation attempt (Fig. 1). After treatment discontinuation, RT-qPCR was performed monthly between month 1–6, every 1.5 months between month 7–12, and every 3 months thereafter. The primary endpoint was the proportion of patients remaining treatment-free by maintaining MMR after 6 and 12 months respectively. Upon molecular relapse, dasatinib therapy was restarted in the previously tolerated dose unless judged inappropriate by the investigator. In these cases, other TKIs were also allowed. Upon molecular relapse, patients were monitored every three months by RT-qPCR. Adverse events were recorded from dasatinib treatment initiation until a minimum of 100 days after the last dose of dasatinib. The study was approved by the ethical review authorities in the participating countries, and was conducted according to the Declaration of Helsinki. This interim analysis was added to the study protocol after study initiation. Reason for adding this interim analysis was that the inclusion of the last patients in the study was delayed due to the COVID19 pandemic leading to substantial deferral of inclusion of the last subject thus also delaying the last visit.

### Definition of molecular responses and molecular relapse

Molecular responses were assessed using RT-qPCR quantification of *BCR::ABL1* mRNA as percentage of control gene expression and expressed as % on the international scale (IS) using a conversion factor in EUTOS certified laboratories. MR levels were defined in accordance with European LeukemiaNet recommendations [15]. In short, in cases of undetectable *BCR::ABL1* levels, ≥10,000 ABL or ≥24,000 GUS, ≥32,000 ABL or ≥77,000 GUS and ≥100,000 ABL or ≥240,000 GUS transcripts, were used as minimal criteria for the definition of MR^4^, MR^4.5^ and MR^5^ respectively. Molecular relapse was defined as *BCR::ABL1* IS% of >0.1% detected by a single test.

### Statistics

Descriptive statistics are presented as proportions with mean or median values and range as a measure of variability. Proportion of patients maintaining MMR without restart of TKI (i.e. in TFR) was calculated using the Kaplan-Meier method with binomial 95% confidence intervals. Patients still in TFR were censored at the date of last follow-up. Statistical comparison between groups were done for exploratory purposes. Based on a previous small study [27] the proportion of patients remaining in MMR after a second TKI discontinuation was postulated to be 30% after 12 months. A 95% confidence interval around this proportion of 20 percentage points was chosen as the desired level of precision, requiring a sample size of 110 patients to be analyzed. However, due to the deferral of inclusion, a smaller sample size and consequently a wider confidence interval was accepted.

## Results

### Patient’s characteristics

**Fig. 2:**
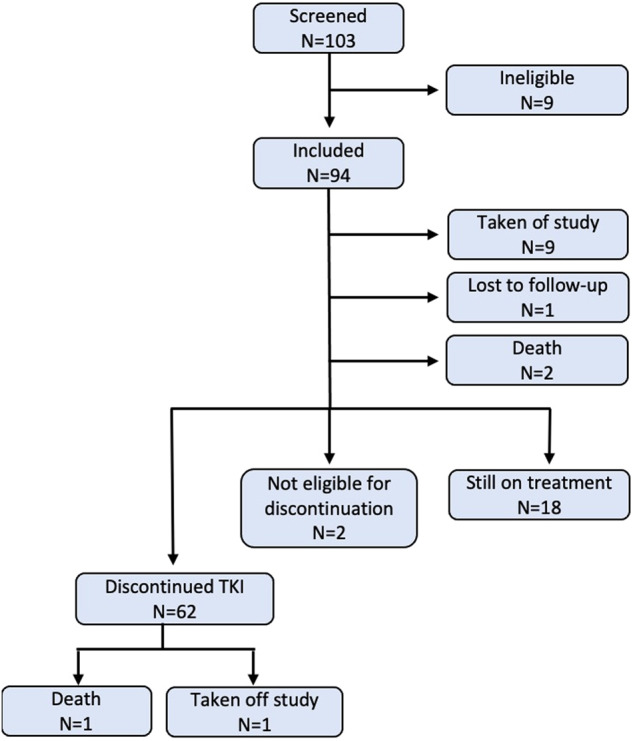
Consort diagram of study progress. Out of 94 included patients, 64 patients had reached 24 months, and 62 were eligible for TKI discontinuation.

Between Oct 2017 and Dec 2021, 103 patients who failed a first TKI discontinuation attempt were screened for participation in the study. Nine patients were deemed ineligible, and 94 patients were included in the study (Fig. 2).

CML history and baseline characteristics for all included patients are described in Table 1. In short, first treatment after CML diagnosis was imatinib, nilotinib and dasatinib in 82% (*n* = 77), 9% (*n* = 8) and 7% (*n* = 7) respectively and treatment information was missing in 2 patients. The median time from diagnosis to the first TKI discontinuation was 74 months (range: 33–228), and median time in MR^4^ prior to the first TKI discontinuation was 41 months (range: 10–156). Sixty percent had performed their prior discontinuation attempt within EURO-SKI, and the median time to relapse after the first stop was 4.5 months (range 1–65). In case of relapse, re-treatment was initiated with either imatinib, nilotinib and dasatinib in 59% (*n* = 55), 13% (*n* = 12) and 27% (*n* = 25) respectively. Information on the type of TKI therapy for molecular relapse was missing in 2 patients. For the patients attempting a second TKI discontinuation (*n* = 62, Table 2), treatment before the first discontinuation attempt was imatinib in 61% (*n* = 38), nilotinib in 15% (*n* = 9), dasatinib in 23% (*n* = 14) and unknown in 2% (*n* = 1).

**Table 1:** Patient characteristics for all included patients.

	All patients included in study (*n* = 94)
Age at diagnosis – years	
Median	49
Range	10–77
Missing values – *n* (%)	1 (1%)
Age at first stop – years	
Median	55
Range	13–82
Missing values – *n* (%)	1 (1%)
Sex – *n* (%)	
Male	48 (51%)
Female	46 (49%)
Missing values – *n* (%)	0 (0%)
ELTS score – *n* (%)	
Low	55 (59%)
Intermediate	15 (16%)
High	5 (5%)
Missing values – *n* (%)	19 (20%)
Time from diagnosis to first stop – months	
Median	74
Range	33–228
Missing values – *n* (%)	1 (1%)
Time in MR4 before first stop – months	
Median	41
Range	10–156
Missing values – *n* (%)	14 (15%)
Participated in EURO-SKI – *n* (%)	
Yes	54 (60%)
No	36 (40%)
Missing values – *n* (%)	4 (4%)
Time from first stop to first loss of MMR - months	
Median	4
Range	1–65
Missing values – *n* (%)	2 (2%)
Time from inclusion to last follow-up – months	
Median	47
Range	1–60
Missing values – *n* (%)	0 (0%)

**Table 2:** Patient characteristics for all patients with a second discontinuation attempt.

	All patients with second stop (*n* = 62)
Age at second stop – years	
Median	60
Range	28–88
Missing values – *n* (%)	0 (0%)
Treatment time from first relapse to second stop - months	
Median	65
Range	36–97
Missing values – *n* (%)	0 (0%)
Time from re-achievement of MR4 to second stop - months	
Median	59
Range	29–91
Missing values – *n* (%)	3 (5%)
Time from second stop to last follow-up – months	
Median	27
Range	1–36
Missing values – *n* (%)	0 (0%)
BCR-ABL level before second stop – *n* (%)	
MR4	11 (18%)
MR4.5	19 (31%)
MR5	32 (52%)
Missing values – *n* (%)	0 (0%)

Twelve patients were taken out of the study in the first 24 months, and hence ineligible for a 2^nd^ stop. Reasons were adverse events (AE) in eight patients, death unrelated to CML in two patients, unknown in one patient and one patient was lost to follow-up. Data were extracted Oct 27^th^ 2022, and at that time, 64 patients had reached 24 months of whom 62 fulfilled discontinuation criteria thus being eligible for a 2^nd^ TKI discontinuation attempt. The median age when attempting the second TKI discontinuation was 60 years (range; 28–88). The median time from re-initiation of TKI therapy to the second discontinuation was 65 months (range; 36–97) and the time from re-achievement of MR^4^ after the first relapse and the second TKI discontinuation was 59 months (range; 29–91).

### Study treatment

At study inclusion, the mean dose of dasatinib was 80 mg (range 20–100) daily for all included patients. For the group of patients who had reached 24 months in the study the mean dasatinib dose was 79 mg (range 20–100) at inclusion and 69 mg (range 20–100) before discontinuation. One patient discontinued dasatinib due to pancreatitis at month 3 and another due to pleural effusion at month 15. Both patients switched treatment to imatinib with maintained molecular response. Both patients were deemed eligible for treatment discontinuation according to the protocol and were kept on study.

### Outcome after the second TKI discontinuation

**Fig. 3:**
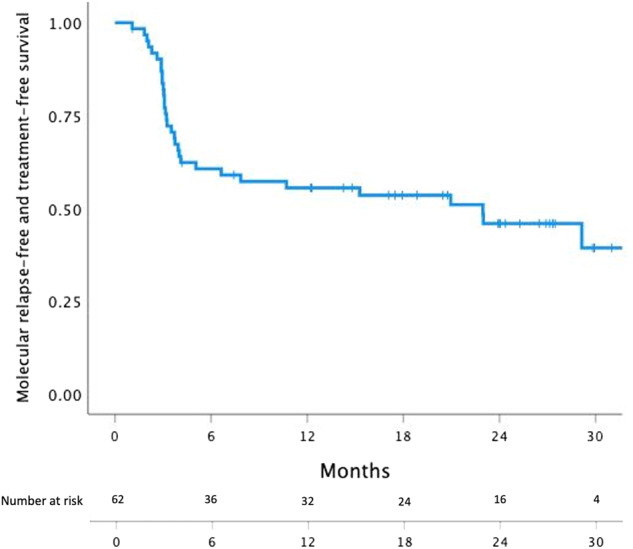
Treatment-free remission (TFR) after second tyrosine kinase inhibitor (TKI) discontinuation. TFR probability at 6, 12 and 24 months was 61, 56 and 46% respectively.

After a median follow-up of 27 months (range, 1–36) from the second TKI discontinuation, 50% (*n* = 31) had re-initiated TKI therapy. TFR was 61% (95% CI; 51–76%), 56% (95% CI; 44–70%), and 46% (95% CI; 36–62%) after 6, 12 and 24 months respectively (Fig. 3). The median time to molecular relapse was 3.2 months (range 1.1–29.1). In comparison the median time to molecular relapse after the first discontinuation attempt had been 4.5 months (range 1.0–64.6). Seventy-one and 84% of the molecular relapses occurred within the first 6 and 12 months, respectively. Five patients had molecular relapse later than 12 months, and two patients had molecular relapse later than 24 months. Among the patients who failed the second stop, 87% (*n* = 27) re-achieved MR^4^ or better, and 97% (*n* = 30) MMR or better after a median follow-up time from molecular relapse of 19 months (range, 1–32). Median time from restart of TKI to MR^4^ re-achievement was 3 months (range 3–9). One patient with molecular relapse one month after discontinuation had BCR-ABL1 of 0.22% on resumed TKI treatment at the last follow-up six months after molecular relapse. One patient died 10 months from TKI discontinuation due to cholangiocarcinoma while still in MR^5^.

Based on the treatment prior to the first discontinuation attempt the proportion of patients failing the 2^nd^ attempt was 18/43 (42%) for imatinib and 12/18 (67%) for 2^nd^ generation TKI after median follow-up of 18 and 17 months from the second discontinuation and median treatment time from the first molecular relapse to the second stop of 63 and 61 months respectively. However, the difference in TFR between the groups did not reach statistical significance (*p* = 0.07, Supplementary Fig. 2)

**Fig. 4:**
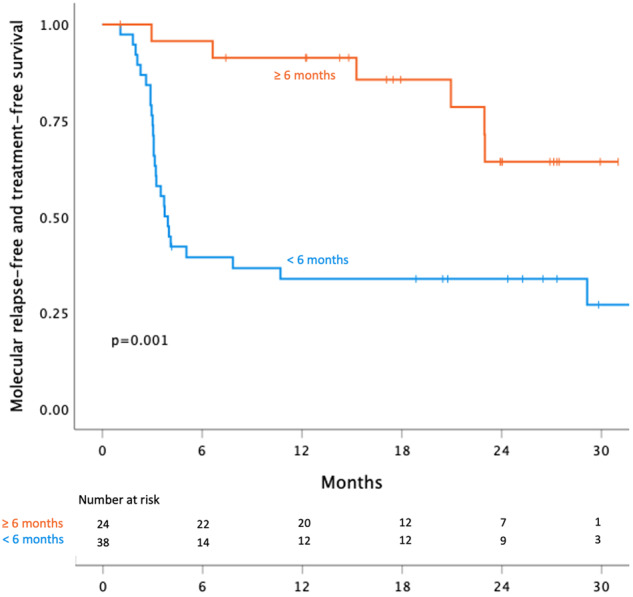
Treatment-free remission (TFR) after second tyrosine kinase inhibitor (TKI) discontinuation by first TFR duration. Patients with a second discontinuation divided by whether the time to molecular relapse after the first discontinuation attempt was more or less than 6 months. The log rank test was used when comparing groups.

The probability of maintaining TFR was higher in patients with a longer first TFR (more than six months) compared to those with a short first TFR (less than six months). Twelve-month TFR success rate was 35% in patients who had a short first TFR compared to 91% in patients with a longer first TFR (*p* = 0.001; see Fig. 4). However, failure rates beyond 12 months were higher in those with a long first TFR. Median time to molecular relapse after the second stop was 3.5 months (range 1.1–29.1) for those with short first TFR versus 18.6 months (range 3.0–24.3) for those with a longer first TFR (*p* = 0.012 using the Man-Whitney U test). The group with a longer first TFR consequently had a higher variance. Among those with less than three months from first discontinuation to first molecular relapse (*n* = 11), the TFR rate at six months was only 13%.

### Adverse events

Adverse events (AE) on study treatment were generally of grade 1-2 and revealed no previously unknown AEs attributable to dasatinib. A summary of AEs is presented in the supplementary material (See Supplementary Table S1). Interestingly, pleural effusions were only seen in three patients (3%), out of which only one was grade 3-4. Two of these patients were treated with dasatinib, and one with imatinib before inclusion in the study. Ten (16%) of the patients attempting a second TKI discontinuation developed musculoskeletal pain after discontinuation, and one (2%) patient had worsening of pre-existing musculoskeletal pain. Serious adverse events reported to be at least possibly related to dasatinib were one case each of pancreatitis, atrial fibrillation, pleural effusion and idiopathic intracranial hypertension. Three patients died during the study, one from sudden cardiac arrest in month six, one from COVID19 in month 21, and one from cholangiocarcinoma in month 33, nine months after TKI discontinuation. All deaths were judged unrelated to dasatinib treatment. Other AEs leading to exclusion of individuals from the study during the first 24 months were pleural effusion (*n* = 2), benign intracranial hypertension (*n* = 1), fatigue (*n* = 1), back pain (*n* = 1), diarrhea (*n* = 1), angina pectoris (*n* = 1) and urosepsis (*n* = 1). In one patient, the reason for exclusion was not clearly stated. In addition, one patient was lost to follow-up after one month.

## Discussion

Interim results of this prospective study of CML patients attempting a second TKI discontinuation after treatment with dasatinib show that 61, 56 and 46% maintained TFR at 6, 12 and 24 months, respectively. Upon re-initiation of TKI therapy 87% regained their previous molecular response and all but one patient had re-achieved at least MMR at the data cut-off time point.

The feasibility and safety of a first TKI treatment discontinuation in patients with CML has been firmly established in previous trials, showing that roughly 50% of patients in sustained DMR will experience a molecular relapse within 12 months after TKI cessation. To what extent these relapsed patients will need to continue TKI treatment for an indefinite time has been largely unknown. TFR rates in our study are encouraging considering that only patients with previous TFR failure were included. Kim et al. reported on high relapse rates after dasatinib re-challenge and a 2^nd^ TKI discontinuation; 94% lost MR4 after a median 3.7 months and were restarted on TKI [28, 29]. This is in sharp contrast to the here presented data, which may at least in part be due to a longer median time in MR^4^ or better. Patients in the TRAD study were treated with dasatinib immediately after molecular relapse, and sustained MR^4^ for one year was sufficient before a second TFR attempt. Similar to data from the first TKI discontinuation, a longer DMR duration prior to a second TKI discontinuation likely translates into improved TFR rates. This data is supported by the French retrospective study RE-STIM, which included patients attempting a second TKI stop in clinical practice and which showed TFR rates after a second stop of 48, 42 and 35% at 12, 24 and 36 months respectively [21]. The NILO post-STIM trial, very similar to the DAstop2 study, evaluated a second TFR in 22 patients treated with the 2^nd^ generation TKI Nilotinib for 24 months [26]. The authors reported TFR rates of 68, 59 and 54% after 12, 24 and 36 months respectively. Notably, in EURO-SKI, the largest study to date investigating a first TKI discontinuation attempt, 80% of molecular relapses occurred within the first 6 months [17]. In RE-STIM 64% of patients had a molecular relapse at last follow-up, and 46% of those occurred later than one year after TKI discontinuation. Similarly, in NILO post-STIM 55% had molecular relapse, and 50% of those occurred after 12 months [26]. This raises the question whether a prolonged period of more intense monitoring might be warranted after a second stop. In our current stop-study, TKI cessation criteria were similar to EURO-SKI (three years of TKI therapy and at least one year of stable MR^4^). At the time of data cut-off, 71 and 84% of molecular relapses had occurred in the first six and 12 months respectively. Notably, two patients had later molecular relapses after more than two years, and it cannot be ruled out that with longer follow-up time the proportion of late molecular relapses may further increase.

The cross-study comparison of TFR rates between different discontinuation trials must be interpreted with some caution due to different treatments, stopping and re-initiation criteria as well as highly variable follow-up times. Previous studies testing a first TKI stop upon initial dasatinib treatment (DASFREE, DADI and D-STOP) were previously reported [30–32]. While DASFREE and DADI used MR^4.5^ as TKI-stop eligibility criterion, D-STOP used MR^4^. TFR rates at 24 months were approximately 46, 55 and 63% in DASFREE, DADI and D-STOP respectively compared with 47% in DAstop2, indicating that a second discontinuation attempt is not necessarily less likely to be successful. Studies with long-term follow-up of TFR rates after a first discontinuation attempt include STIM, A-STIM and ENESTfreedom presenting TFR rates of 38, 46 and 43% after 5, 7 and 5 years respectively [4, 33, 34]. Relapse rates beyond 36 months after first TKI discontinuation have been assessed in A-STIM and EURO-SKI, showing that approximately 10% of patients in TFR at 36 months experience late molecular relapse [33, 35]. Long-term data is essential in order to draw conclusions regarding the factors associated with molecular relapse, and the incidence of late relapses after a second TKI discontinuation.

We hypothesized that re-treatment with a second generation TKI might be beneficial when entering a second TFR phase, especially for those with a previous discontinuation after imatinib treatment. Relapse rates in our analysis were 42 and 67% for patients treated with imatinib versus 2^nd^ generation TKI just before the first discontinuation attempt. This suggests that patients with a previously unsuccessful long-term TFR on imatinib are more likely to achieve TFR upon 2^nd^ TKI stop when compared to patients who failed their previous attempt after therapy with a 2^nd^ generation TKI. However, sample sizes in our study are too small to draw any robust conclusions on this subject. We suggest that with more patients attempting a second TKI stop, the impact of TKI switch from imatinib to a second generation TKI between the first and the second discontinuation attempt should be defined in more detail.

Similar to findings in RE-STIM where patients with more than three months to first relapse had significantly less relapses after the second TKI discontinuation, patients experiencing their molecular relapse later than six months upon the first TKI cessation were less likely to relapse in DAstop2. Accordingly, the median time to the second molecular relapse was significantly longer in the patients with a late (>6 months) first molecular relapse (18.6 versus 3.5 months). Nevertheless, after 12 months the gap between the survival curves for patients with early versus late (<versus > 6 months) first molecular relapse decreased due to a higher number of late relapses in the latter group. Once again, this highlights the need for prolonged monitoring within studies or registries in order to determine whether duration of first TFR is indeed a prognostic factor, or if it is merely a marker of different BCR-ABL1 kinetics. In addition, other (i.e. immunologic, inflammatory and genetic) biomarkers are needed that may help to better predict the likelihood of a successful 2^nd^ TFR attempt.

In summary, the interim results from our study testing a second discontinuation attempt after re-treatment with dasatinib in CML-CP show TFR rates comparable to previous studies testing a first TFR attempt. Longer time to relapse after a first TKI discontinuation appears to be correlated to a better chance of maintaining TFR upon 2^nd^ TKI discontinuation, and variables associated with a successful second TKI discontinuation will be further explored in the final report of this study.

### Supplementary information

Supplementary figure legends

Supplementary figure 1

Supplementary figure 2

Table S1

Table S2

Table S3

## Data Availability

The datasets generated and analyzed during the current study are not publicly available due to the integrity of study subjects but are available from the corresponding author on reasonable request.
