# Correction: Treatment-free remission after a second TKI discontinuation attempt in patients with Chronic Myeloid Leukemia re-treated with dasatinib – interim results from the DAstop2 trial

**DOI:** 10.1038/s41375-024-02184-z

**Published:** 2024-02-28

**Authors:** Hjalmar Flygt, Stina Söderlund, Johan Richter, Susanne Saussele, Perttu Koskenvesa, Leif Stenke, Satu Mustjoki, Andreja Dimitrijevic, Jesper Stentoft, Waleed Majeed, Lydia Roy, Dominik Wolf, Arta Dreimane, Bjørn Tore Gjertsen, Tobias Gedde-Dahl, Erik Ahlstrand, Berit Markevärn, Henrik Hjorth-Hansen, Jeroen Janssen, Ulla Olsson-Strömberg

**Affiliations:** 1 Department of Medical Science and Division of Hematology, Uppsala University Hospital, Uppsala, Sweden; 2 Department of Hematology, Oncology and Radiation Physics, Skåne University Hospital, Lund, Sweden; 3 Medical Clinic, Medical Faculty Mannheim of the University of Heidelberg, Mannheim, Germany; 4 Department of Hematology, Hematology Research Unit Helsinki and Helsinki University Hospital Comprehensive Cancer Center, Helsinki, Finland; 5 Department of Hematology, Karolinska University Hospital and Department of Medicine, Karolinska Institutet, Stockholm, Sweden; 6 Translational Immunology Research Program and Department of Clinical Chemistry and Hematology, University of Helsinki, Helsinki, Finland; 7 ICAN Digital Precision Cancer Medicine Flagship, Helsinki, Finland; 8 Department of Hematology, Odense University Hospital, Odense, Denmark; 9 Department of Hematology, Aarhus University Hospital, Aarhus, Denmark; 10 Department of Hemato-Oncology, Stavanger University Hospital, Stavanger, Norway; 11 French CML group Fi-LMC, Centre Léon Bérard, Lyon, Hôpital Universitaire Henri Mondor, AP-HP, Service d’hématologie Clinique & Faculté de Santé, Université Paris Est Créteil, Créteil, France; 12 Department of Hematology and Oncology, Comprehensive Cancer Center Innsbruck (CCCI), Tyrolean Cancer Research Institute (TKFI), Medical University Innsbruck, Innsbruck, Austria; 13 Medical Clinic 3, Universitätsklinikum, Bonn, Germany; 14 Department of Hematology, Linköping University Hospital, Linköping, Sweden; 15 Department of Internal Medicine, Hematology Section, Haukeland University Hospital, Bergen, Norway; 16 Department of Hematology, Oslo University Hospital, Rikshospitalet, Oslo, Norway; 17 Department of Medicine, Faculty of Medicine and Health, Örebro University, Örebro, Sweden; 18 Department of Hematology, Umeå University Hospital, Umeå, Sweden; 19 Department of Hematology, St. Olavs Hospital, Trondheim, Norway; 20 Department of Hematology, Radboud University medical center, Nijmegen, The Netherlands

**Keywords:** Myeloproliferative disease, Targeted therapies

Correction to: *Leukemia* 10.1038/s41375-024-02145-6 published online 26 January 2024

The information provided in the Figure III legend states:

“TFR probability at 6, 12 and 24 months was 62, 55 and 42%respectively”, the figure should have appeared as shown below:

“TFR probability at 6, 12 and 24 months was 61, 56 and 46%, respectively”

