# Supplementary material for: Treatment-free remission after a second TKI discontinuation attempt in patients with Chronic Myeloid Leukemia re-treated with dasatinib – interim results from the DAstop2 trial
Source: Leukemia. 2024 Jan 26;38(4):781–7. doi: 10.1038/s41375-024-02145-6 (PMC10997502; doi:10.1038/s41375-024-02145-6)
Supplement: Supplementary file 1 — Supplementary figure legends [file 41375_2024_2145_MOESM1_ESM.pdf]

Supplementary figure 1 – ***Tyrosine kinase inhibitor treatment for all included patients at diagnosis, first discontinuation, first molecular relapse, and at inclusion in DAstop 2.***

Supplementary figure 2 - ***Treatment-free remission (TFR) in patients with a second tyrosine kinase inhibitor discontinuation (TKI) and patients divided by whether treatment was imatinib or 2nd generation TKI prior to the first discontinuation attempt. The log rank test was used when comparing groups.***
