# Supplementary figures and images for: Treatment-free remission after a second TKI discontinuation attempt in patients with Chronic Myeloid Leukemia re-treated with dasatinib – interim results from the DAstop2 trial
Source: Leukemia. 2024 Jan 26;38(4):781–7. doi: 10.1038/s41375-024-02145-6 (PMC10997502; doi:10.1038/s41375-024-02145-6)

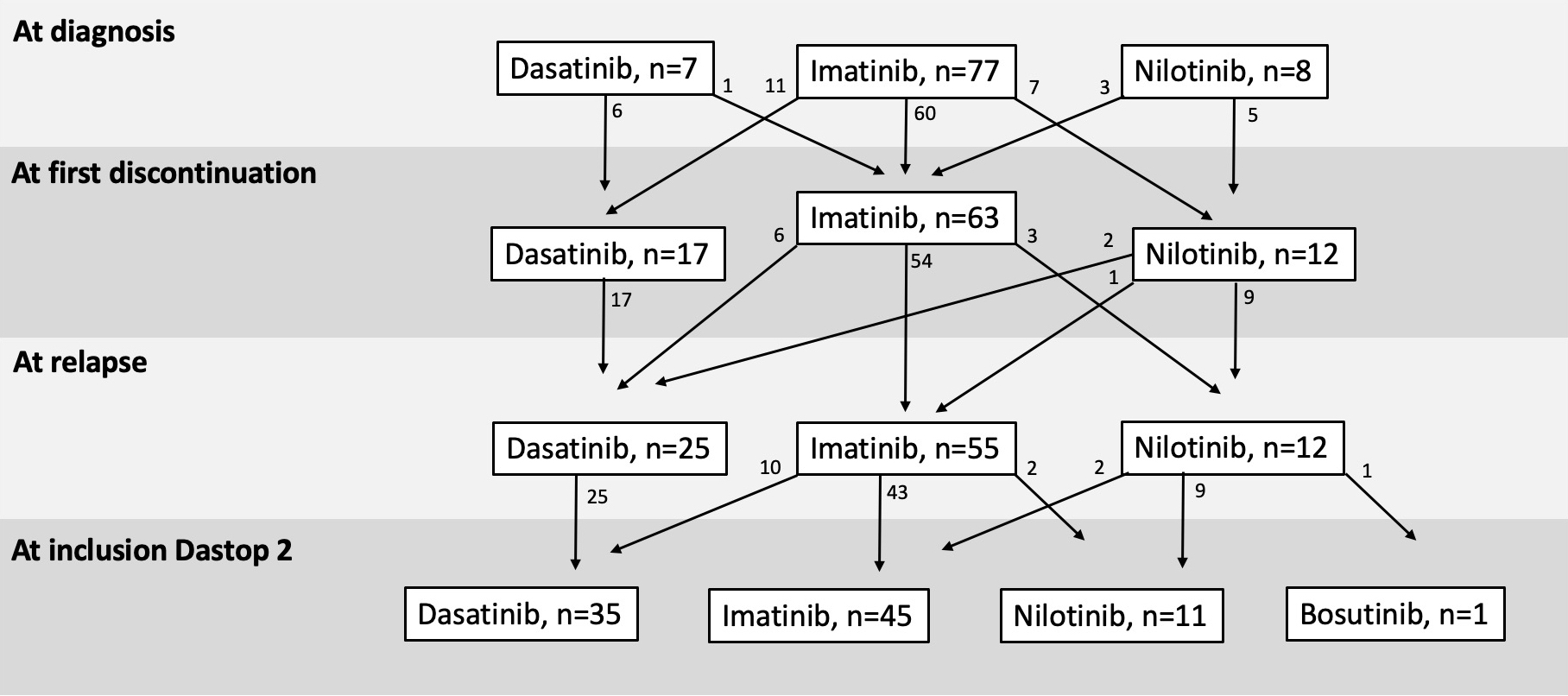

Supplement: Supplementary file 2 — Supplementary figure 1 [file 41375_2024_2145_MOESM2_ESM.jpg]

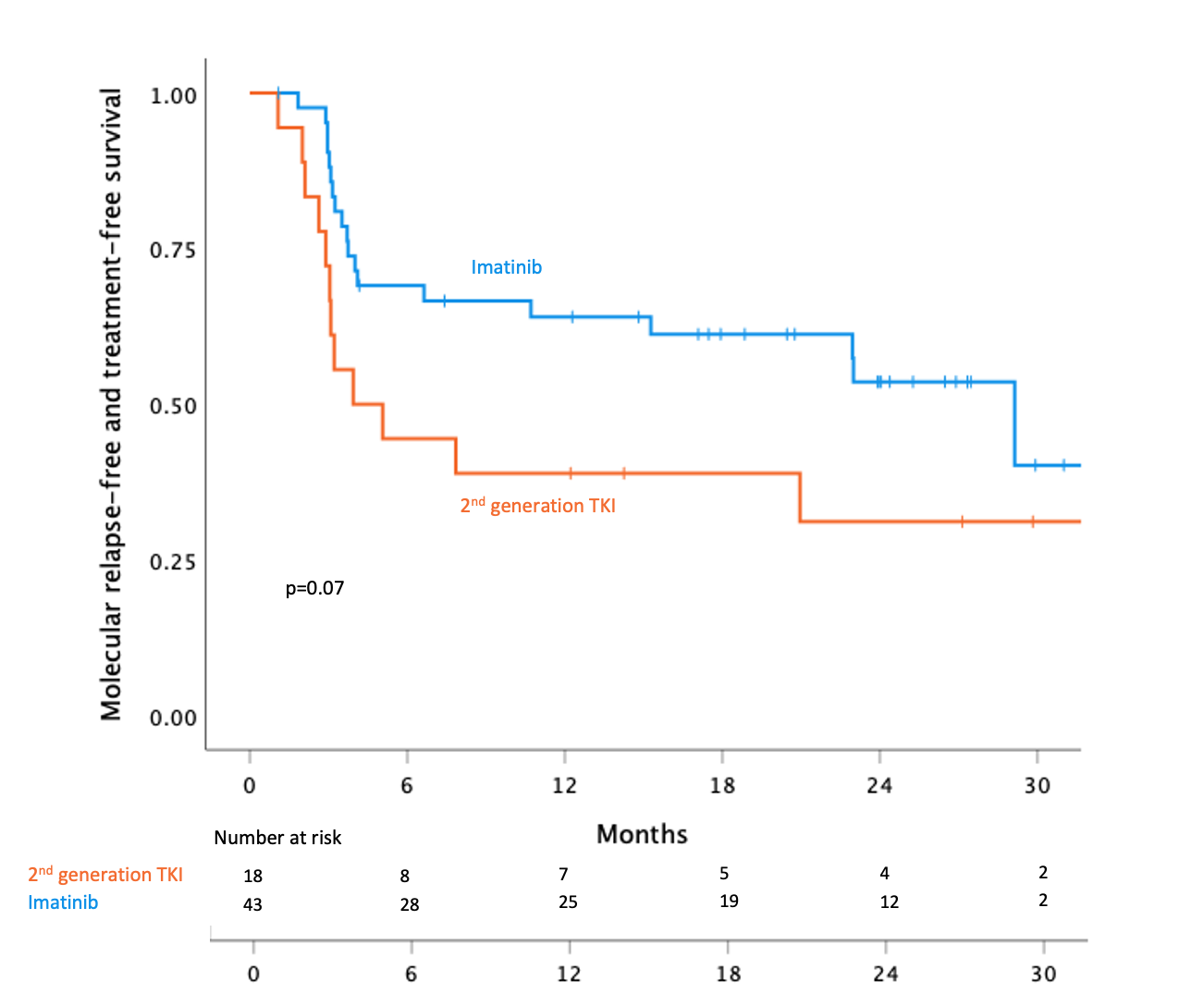

Supplement: Supplementary file 3 — Supplementary figure 2 [file 41375_2024_2145_MOESM3_ESM.tif]
